# Smart Grid Outlier Detection Based on the Minorization–Maximization Algorithm

**DOI:** 10.3390/s23198053

**Published:** 2023-09-24

**Authors:** Lina Qiao, Wengen Gao, Yunfei Li, Xinxin Guo, Pengfei Hu, Feng Hua

**Affiliations:** 1 College of Electrical Engineering, Anhui Polytechnic University, Wuhu 241000, China; qln1017@163.com (L.Q.); lyf@mail.ahpu.edu.cn (Y.L.); guoxinxin@ahpu.edu.cn (X.G.); h382024188@163.com (P.H.); huafeng980525@163.com (F.H.); 2 Key Laboratory of Advanced Perception and Intelligent Control of High-End Equipment, Chinese Ministry of Education, Wuhu 241000, China

**Keywords:** outlier detection, the Minorization–Maximization algorithm, localization, power system

## Abstract

Outliers can be generated in the power system due to aging system equipment, faulty sensors, incorrect line connections, etc. The existence of these outliers will pose a threat to the safe operation of the power system, reduce the quality of the data, affect the completeness and accuracy of the data, and thus affect the monitoring analysis and control of the power system. Therefore, timely identification and treatment of outliers are essential to ensure stable and reliable operation of the power system. In this paper, we consider the problem of detecting and localizing outliers in power systems. The paper proposes a Minorization–Maximization (MM) algorithm for outlier detection and localization and an estimation of unknown parameters of the Gaussian mixture model (GMM). To verify the performance of the method, we conduct simulation experiments by simulating different test scenarios in the IEEE 14-bus system. Numerical examples show that in the presence of outliers, the MM algorithm can detect outliers better than the traditional algorithm and can accurately locate outliers with a probability of more than 95%. Therefore, the algorithm provides an effective method for the handling of outliers in the power system, which helps to improve the monitoring analyzing and controlling ability of the power system and to ensure the stable and reliable operation of the power system.

## 1. Introduction

With the rapid development of the power industry around the world, the scale of the power system has gradually expanded, and its operation mode and network structure have become more complex. The combination of power grids and modern information forms smart grids, which pose greater challenges to the safety and reliability of power system operations. In order to improve the operational efficiency and reliability of the power grid, the dispatching system needs to collect complete and reliable real-time data for processing to facilitate online analysis and decision control of advanced application software.

Power system state estimation is one of the core functions of the Energy Management System (EMS) in the power system dispatching center [1,2,3]. Its function refers to the control center to collect various measurement data through sensors and to estimate the current operating state of the power system according to the measurement data. The safe and economical operation of modern power grids depends on the EMS. The many functions of the Energy Management System can be divided into two parts: online application for real-time change analysis of the power grid and offline application for typical power flow section analysis. Power system state estimation is the core of power system online monitoring, analysis, and control functions and plays an important role in the intelligent analysis of power grid dispatching. State estimation is the basis of most advanced software for online applications, and the accuracy of the state estimation results is closely related to the accuracy of the subsequent analysis calculation results. Due to the continuous improvement in the automation of the power system, the quality requirements for real-time measurement data in state estimation are becoming higher and higher. In addition to the measurement noise, there may be outliers in the obtained measurement data. Outliers [4,5,6] can be caused by a variety of factors. The following are some common causes of outliers:

(1) Sensors can malfunction or fail, resulting in inaccurate or incomplete data collection, resulting in outliers.
(2) Human factors, such as incorrect data entry, operational errors, etc., may cause data anomalies.
(3) Natural disasters and emergencies, such as storms, earthquakes, fires, explosions, etc., may cause damage to electric power facilities and thus generate outliers.
(4) Factors such as power equipment failure, damage, data transmission, and processing errors can lead to outliers.

The existence of outliers will cause the state estimation results to deviate significantly from the actual operation of the system, and the performance of the estimator will be seriously degraded, so the work of outlier detection becomes particularly important.

To solve the problem of outliers in measurement data, the traditional detection methods include weighted least squares (WLS) [7,8,9], residual search method [10,11], and non-quadratic criterion method [12,13]. These methods are further elaborated on below. The basic idea of the WLS is to adjust the weights of measurements by assigning larger weights to relatively reliable and accurate measurements and smaller weights to measurements that are relatively unreliable and affected by outliers. By adjusting the weights, the WLS algorithm can reduce the effect of outliers on the fitted results and improve the accuracy of the parameter estimates. However, it is important to note that the WLS algorithm has some limitations when dealing with outliers. When the number of outliers is high or the difference between the outliers and normal values is large, the WLS algorithm may not be able to eliminate the effect of the outliers. The residual search method uses weighted residuals and standard residuals to sort the measurement data. After eliminating the data with large residuals, the state estimation is re-estimated to achieve the purpose of optimal estimation. However, the disadvantage of this method is that the calculation amount is large, and it is easy to have residual pollution and residual submergence, resulting in false detection or missed detection. The strategy adopted by the non-quadratic criterion method is to successively reduce the weight of suspicious data, rather than eliminating suspicious data one by one. Re-estimation is avoided to reduce the amount of calculation. However, this may lead to difficult consequences of convergence, and there is no guarantee that the final estimate will be optimal. In summary, traditional outlier detection algorithms have certain limitations in addressing outlier problems. Therefore, new methods have been proposed to solve the problem of the presence of outliers.

In this paper, the Minorization–Maximization (MM) algorithm [14,15,16,17,18,19,20] is used to detect outliers. Compared with traditional methods for detecting outliers, the MM algorithm can detect outliers more accurately, especially for complex data distributions, and can obtain better performance. The MM algorithm is an optimization algorithm whose basic idea is to optimize the original function to be optimized indirectly by optimizing the alternative function by finding an easier function to be optimized. In each iteration, the algorithm optimizes the original function by transforming the original problem into a more tractable problem through a series of derivations and transformations. The algorithm first appeared in the field of online search in the 1970s [21], when Ortega and Rheinboldt first mentioned the MM principle [22]. Later, de Leeuw proposed the first MM algorithm in a multidimensional scale analysis, and Hunter and Lange named it the MM algorithm [23], which has since been widely used in statistics. MM algorithms are an important tool in optimization problems because of their conceptual simplicity, ease of implementation, and numerical stability. Moreover, MM algorithms are provably convergent optimization algorithms that are guaranteed to find either a globally optimal solution or a locally optimal solution under certain conditions. Overall, the MM algorithm is a very important optimization algorithm with a wide range of application scenarios and excellent properties that can effectively solve many practical problems.

This paper is divided into six main sections. First, in Section 2, the normal measurement model and the measurement model containing outliers are described. Then, Section 3 introduces the basic principles of the MM algorithm, including the iterative process of the MM algorithm, the objective function, and its optimization methods. In Section 4, this paper further introduces the parameter estimation based on the MM algorithm, including the application of the MM algorithm in anomaly detection and the specific implementation method of parameter estimation. In Section 5, the convergence and complexity of the algorithm are analyzed. In Section 6, the simulation results analysis of this paper are given, and the superiority and feasibility of the anomaly detection method based on the MM algorithm are proved through a detailed analysis of the experimental results. Finally, in Section 7, the paper is summarized.

## 2. System Model

### 2.1. Measurement Model

The measurement vector z is a nonlinear function [24,25] of the state vector x and can be expressed as

(1) z=h(x)+e

where $e={\left[e_{1},e_{2},\ldots ,e_{m}\right]}^{T}\in R^{m\times 1}$ is the measurement error vector, and e is assumed to be the Gaussian measurement noise with zero mean and covariance $\sigma^{2}$, i.e., $e∼N(0,\sigma^{2})$. $h={\left[h_{1}\left(x\right),h_{2}\left(x\right),\ldots ,h_{m}\left(x\right)\right]}^{T}\in R^{m\times 1}$, $h_{i}\left(x\right)$ is a nonlinear function relating the ith measurement to the state vector x. An alternative expression for the nonlinear relationship h(·) between the state vector and the measurement vector can be given by the following:

(2) $$P_{i}=V_{i}\sum_{j\in \Omega_{i}}^{}V_{j}\left(G_{ij}\cos\theta_{ij}+B_{ij}\sin\theta_{ij}\right)$$

(3) $$Q_{i}=V_{i}\sum_{j\in \Omega_{i}}^{}V_{j}\left(G_{ij}\sin\theta_{ij}-B_{ij}\cos\theta_{ij}\right)$$

(4) $$P_{ij}=V_{i}^{2}\left(g_{si}+g_{ij}\right)-V_{i}^{}V_{j}^{}\left(g_{ij}\cos\theta_{ij}+b_{ij}\sin\theta_{ij}\right)$$

(5) $$Q_{ij}=-V_{i}^{2}\left(b_{si}+b_{ij}\right)-V_{i}^{}V_{j}^{}\left(g_{ij}\sin\theta_{ij}-b_{ij}\cos\theta_{ij}\right)$$

In this expression, $P_{i}$ and $Q_{i}$ denote the active and reactive power injection at bus *i*, respectively; $P_{ij}$ and $Q_{ij}$ denote the real and reactive power flow from bus *i* to bus *j*, respectively; $V_{i}$ represents the voltage at bus *i*; $\theta_{i}$ denotes the phase angle at bus *i*; $\theta_{ij}$ denotes the phase difference between buses *i* and *j*; $G_{ij}+jB_{ij}$ represents the line conductance between buses *i* and *j*; $g_{ij}+jb_{ij}$ represents the conductance of the branch branch of bus *i*; and $\Omega_{i}$ is the set of buses associated with bus *i*.

In power system SE, the state vector, x, is usually composed of all nodes voltage amplitudes and their corresponding angles. The measurement vector, z, consists of active and reactive power injection and flow, voltage, and current magnitudes obtained from SCADA.

The estimation of the state vector x can be iteratively solved using weighted least squares (WLS); then,

(6) $$x^{k+1}=x^{k}+{\left[H^{kT}R^{-1}H^{k}\right]}^{-1}H^{kT}R^{-1}\left(z-h\left(x^{k}\right)\right)$$

where $x^{k}$ is the solution vector at the kth iteration; $R=E[e\cdot e^{T}]$ is the measurement error covariance matrix; and H=(∂h(x)/∂x) is the measurement Jacobian matrix, that is

(7) $$H\left(x\right)=\left[\begin{array}{c} \frac{\partial P_{i}}{\partial \theta }\;\frac{\partial P_{i}}{\partial V}\\ \frac{\partial Q_{i}}{\partial \theta }\;\frac{\partial Q_{i}}{\partial V}\\ \frac{\partial P_{ij}}{\partial \theta }\;\frac{\partial P_{ij}}{\partial V}\\ \frac{\partial Q_{ij}}{\partial \theta }\;\frac{\partial Q_{ij}}{\partial V} \end{array}\right]$$

The weighted least squares iterative algorithm is a common method that is used to estimate state vectors.

### 2.2. Measurement Model with Outliers

If the observations completely conform to the measurement model in (1), the estimation $\hat{x}$ can be obtained using WLS. However, when a few outliers exist in the measurement, the estimation performance may be seriously degraded. Outliers are deviations or anomalies in data that can be caused by a variety of factors, including sensor failures, communication or human errors, power equipment failures, damages, and errors in data transmission or processing. In the presence of outliers, the observations do not follow the model in (1) exactly, but they can obey the following model:

(8) z=h(x)+a+e

where the vector a denotes the outliers contained in the observations.

Traditional outlier detection methods usually use normalized residuals obtained based on weighted least squares to determine the presence of outliers in the data. However, this method has limitations in facing residual contamination and residual flooding problems. To overcome these problems, some improved outlier detection methods can be considered.

### 2.3. Gaussian Mixture Model for Measurements

Suppose that there are *S* outliers for *M* measurements of the measurement vector *z*. In the measurement acquisition process, we perform outlier detection by acquiring L(L≥1) measurement vectors to determine whether there are outliers in the measurements. In the presence of outliers, the noise characteristics of the data are altered. Therefore, the *i*th measurement of the *l*th measurement vector $z_{l}$ is denoted as follows:

(9) $$z_{i,l}=\left\{\begin{array}{c} h_{i,l}\left(x\right)+e_{i,l,1}\\ h_{i,l}\left(x\right)+e_{i,l,2} \end{array}\right.$$

where $e_{i.l,1}$ and $e_{i.l,2}$ obey the Gaussian distribution, respectively, i.e., $e_{i.l,1}∼N\left(\mu_{1},\sigma_{1}^{2}\right)$ and $e_{i.l,2}∼N\left(\mu_{2},\sigma_{2}^{2}\right)$. $e_{i.l,1}$ denotes the measurement error vector in the absence of outliers. $e_{i.l,2}$ denotes the measurement error vector in the presence of outliers. Hence, the Gaussian mixture model (GMM) [26,27,28] is introduced to represent the probability density of e, and p(e) is given by

(10) $$p\left(e\right)=\sum_{k=1}^{2}\pi_{k}N\left(e|\mu_{k},\sigma_{k}^{2}\right)$$

where $N(e|\mu_{k},\sigma_{k}^{2})$ denotes the *k*th component in the mixture model and $\pi_{k}$ is the mixture coefficient and satisfies

(11) $$\sum_{k=1}^{2}\pi_{k}=1,\;0\leq \pi_{k}\leq 1$$

It is assumed that *M* represents the number of measurements and *S* represents the number of outliers, hence $\pi_{1}=\left(M-S\right)/M$, $\pi_{2}=S/M$. The parameters that need to be estimated in GMM are $\theta ={\left[\pi_{1},\pi_{2},\mu_{1},\mu_{2},\sigma_{1}^{2},\sigma_{2}^{2}\right]}^{T}$. Then, the objective function of θ can be written as

(12) $$\begin{array}{c} J(\theta ,(z-h(x)))\\ =\ln p((z-h(x));\theta )\\ =\ln(\sum_{k=1}^{2}\pi_{k}N\left(\left(z-h\left(x\right)\right)|\mu_{k},\sigma_{k}^{2}\right))\\ =\sum_{i=1}^{M}\sum_{l=1}^{L}\ln(\sum_{k=1}^{2}\pi_{k}N(\left(z_{i,l}-h_{i,l}\left(x\right)\right)|\mu_{k},\sigma_{k}^{2})) \end{array}$$

When unknown parameters are estimated, we cannot use the maximum likelihood method to derive the parameters that maximize the likelihood function as the single Gaussian model (SGM) does. To solve this problem, the Minorization–Maximization (MM) algorithm can be used. Through the MM algorithm, we can iteratively calculate the parameters in GMM.

## 3. The Minorization–Maximization Algorithm

For all observation data, it is not known in advance which sub-distribution they belong to. Each submodel has unknown parameters, and direct derivation cannot be calculated. It needs to be solved with an iterative approach. Therefore, the Minorization–Maximization algorithm can be used to solve the problem that unknown parameters are difficult to solve.

The MM algorithm is an iterative approach. The basic idea is to find a function that is easier to optimize as a surrogate function for the objective function and to indirectly optimize the objective function by optimizing the surrogate function. In each iteration, a new alternative function is constructed based on the parameter estimates obtained from the previous iteration. The new substitution function is then optimized to obtain the parameter estimate in this iteration and to use it in the calculation of the next iteration. Through continuous iteration, the estimated value of the parameter constantly approaches the optimal solution of the objective function.

According to the basic idea of MM algorithm, the surrogate function should satisfy

(13) $$\left\{\begin{array}{c} Q\left(\theta |\theta^{\left(s\right)}\right)\leq J\left(\theta ,\left(z-h\left(x\right)\right)\right)\\ Q\left(\theta^{\left(s\right)}|\theta^{\left(s\right)}\right)=J\left(\theta^{\left(s\right)},\left(z-h\left(x\right)\right)\right) \end{array}\right.$$

where $\theta^{\left(s\right)}$ denotes the *s*th iteration of θ and the surrogate function $Q(\theta |\theta^{\left(s\right)})$ is always below the objective function J(θ,z). When $\theta =\theta^{\left(s\right)}$, the surrogate function $Q(\theta |\theta^{\left(s\right)})$ is tangent to the function J(θ,z). Then, the surrogate function $Q(\theta |\theta^{\left(s\right)})$ is maximized to obtain

(14) $$\theta^{\left(s+1\right)}=\underset{\theta }{\arg\max}\;Q\left(\theta |\theta^{\left(s\right)}\right)$$

as the (s+1)th iteration of the θ. Finally, the maximum likelihood algorithm is used to estimate the unknown parameters.

From the basic principles of the MM algorithm, the difficulty of using the MM algorithm to estimate unknown parameters is in constructing a suitable function as a surrogate function of the objective function. The construction of the surrogate function is described in the next section.

## 4. Parameter Estimation of GMM

Constructing a suitable surrogate function is the key to estimating unknown parameters using the MM algorithm. Tian et al. [17] propose a new Assembly and Decomposition method (AD) to construct the surrogate function. Among them, technology is the basis of the MM algorithm, which guides the construction of the surrogate function. The D technique decomposes the objective function and then optimizes it.

### 4.1. Construction of Surrogate Function

According to the AD method, Jensen’s inequality is used to construct a surrogate function in the minorization step. Jensen’s inequality is given by the Equation (10), that is,

(15) $$\varphi \left(\sum_{i=1}^{n}\alpha_{i}x_{i}\right)\geq \sum_{i=1}^{n}\alpha_{i}\varphi (x_{i})$$

where we let φ(·) be a concave function, $\alpha_{i}\geq 0$, and $\sum_{i=1}^{n}\alpha_{i}=1$. Therefore, the surrogate function is expressed as

(16) $$\begin{array}{cc} Q(\theta |\theta^{\left(s\right)}) & =\sum_{i=1}^{M}\sum_{l=1}^{L}\left[\sum_{k=1}^{2}w_{i,l,k}\ln\frac{\pi_{k}N(\left(z_{i,l}-h_{i,l}\left(x\right)\right)|\mu_{k},\sigma_{k}^{2})}{w_{i,l,k}}\right]\\ \; & =\sum_{i=1}^{M}\sum_{l=1}^{L}\sum_{k=1}^{2}w_{i,l,k}\ln(\pi_{k}N(\left(z_{i,l}-h_{i,l}\left(x\right)\right)|\mu_{k},\sigma_{k}^{2})+c^{\left(s\right)} \end{array}$$

where $\theta^{\left(s\right)}$ represents the *s*th iteration of θ and the weight function $w_{i,l,k}$ is denoted as

(17) $$w_{i,l,k}=\frac{\pi_{k}^{\left(s\right)}N\left(\left(z_{i,l}-h_{i,l}\left(x\right)\right)|\mu_{k}^{\left(s\right)},\sigma_{k}^{2,(s)}\right)}{p(\left(z_{i,l}-h_{i,l}\left(x\right)\right);\theta_{}^{\left(s\right)})}$$

where

(18) $$p\left(\left(z_{i,l}-h_{i,l}\left(x\right)\right);\theta_{}^{\left(s\right)}\right)=\sum_{k=1}^{2}\pi_{k}N\left(\left(z_{i,l}-h_{i,l}\left(x\right)\right)|\mu_{k}^{\left(s\right)},\sigma_{k}^{2,(s)}\right)$$

Define $\Phi_{i,l,k}^{\left(s\right)}=\pi_{k}^{\left(s\right)}N\left(\left(z_{i,l}-h_{i,l}\left(x\right)\right)|\mu_{k}^{\left(s\right)},\sigma_{k}^{2,(s)}\right)$. By comparing the values of $\Phi_{i,l,k}^{\left(s\right)}$, the following weight function is given.

(19) $$w_{i,l}^{\left(s\right)}=\left\{\begin{array}{c} 1,\;\Phi_{i,l,1}^{\left(s\right)}\geq \Phi_{i,l,2}^{\left(s\right)}\\ 0,\;\Phi_{i,l,1}^{\left(s\right)}<\Phi_{i,l,2}^{\left(s\right)} \end{array}\right.$$

The weight function satisfies

(20) $$w_{i,l,k}\geq 0,\sum_{k=1}^{2}w_{i,l,k}=1$$

and

(21) $$c^{\left(s\right)}=-\sum_{i=1}^{M}\sum_{l=1}^{L}\sum_{k=1}^{2}w_{i,l,k}\ln w_{i,l,k}$$

is a constant term independent of parameter θ.

### 4.2. Parameter Estimation of $\pi_{k}$, $\mu_{k}$, and $\sigma_{k}^{2}$

The surrogate function in Equation (9) can be decomposed as

(22) $$\begin{array}{c} Q\left(\theta |\theta^{\left(s\right)}\right)=\sum_{i=1}^{M}\sum_{l=1}^{L}\sum_{k=1}^{2}w_{i,l,k}\ln\pi_{k}+\sum_{i=1}^{M}\sum_{l=1}^{L}\sum_{k=1}^{2}w_{i,l,k}\ln\left(N\left(\left(z_{i,l}-h_{i,l}\left(x\right)\right)|\mu_{k},\sigma_{k}^{2}\right)\right)+c^{\left(s\right)} \end{array}$$

where

(23) $${J_{1}}^{\left(s\right)}\left(\pi_{k}\right)=\sum_{i=1}^{M}\sum_{l=1}^{L}\sum_{k=1}^{2}w_{i,l,k}\ln\pi_{k}$$

(24) $$J_{2}^{\left(s\right)}\left(\mu_{k},\sigma_{k}^{2}\right)=\sum_{i=1}^{M}\sum_{l=1}^{L}\sum_{k=1}^{2}w_{i,l,k}\ln\left(N\left(\left(z_{i,l}-h_{i,l}\left(x\right)\right)|\mu_{k},\sigma_{k}^{2}\right)\right)$$

In the (s+1)th iteration, the maximum likelihood estimation (MLE) of parameter θ can be constructed by maximizing the surrogate function, that is

(25) $$\theta^{\left(s+1\right)}=\underset{\theta }{\arg\max}\;Q\left(\theta |\theta^{\left(s\right)}\right)$$

The expansion of Equation (24) can be written as

(26) $$\begin{array}{cc} \; & J_{2}^{\left(s\right)}\left(\mu_{k},\sigma_{k}^{2}\right)\\ & =\sum_{i=1}^{M}\sum_{l=1}^{L}\left[w_{i,l,1}\ln\left(N\left(\left(z_{i,l}-h_{i,l}\left(x\right)\right)|\mu_{1},\sigma_{1}^{2}\right)\right)+w_{i,l,2}\ln\left(N\left(\left(z_{i,l}-h_{i,l}\left(x\right)\right)|\mu_{2},\sigma_{2}^{2}\right)\right)\right]\\ \; & =\sum_{i=1}^{M}\sum_{l=1}^{L}[w_{i,l,1}\left(\ln\frac{1}{{\left(2\pi \sigma_{1}^{2}\right)}^{\frac{N}{2}}}-\frac{{\left(z_{i,l}-h_{i,l}\left(x\right)-\mu_{1}\right)}^{2}}{2\sigma_{1}^{2}}\right)+w_{i,l,2}\left(\ln\frac{1}{{\left(2\pi \sigma_{2}^{2}\right)}^{\frac{N}{2}}}-\frac{{\left(z_{i,l}-h_{i,l}\left(x\right)-\mu_{2}\right)}^{2}}{2\sigma_{2}^{2}}\right)] \end{array}$$

The partial derivative of the surrogate function with respect to unknown parameters can be obtained

(27) $$\frac{\partial }{\partial \pi_{k}}\left[J_{1}^{\left(s\right)}\left(\pi_{k}\right)\right]=0$$

(28) $$\frac{\partial }{\partial \mu_{k}}\left[{J_{2}}^{\left(s\right)}\left(\mu_{k},\sigma_{k}^{2}\right)\right]=0$$

(29) $$\frac{\partial }{\partial \sigma_{k}^{2}}\left[{J_{2}}^{\left(s\right)}\left(\mu_{k},\sigma_{k}^{2}\right)\right]=0$$

and an iterative formula for the parameters can be obtained by solving the above equation, as follows:

(30) $$\pi_{k}^{\left(s+1\right)}=\frac{\sum_{i=1}^{M}\sum_{l=1}^{L}w_{i,l,k}}{ML}$$

(31) $$\mu_{k}^{\left(s+1\right)}=\frac{\sum_{i=1}^{M}\sum_{l=1}^{L}\left(z_{i,l}-h_{i,l}\left(x^{\left(s\right)}\right)\right)w_{i,l,k}}{\sum_{i=1}^{M}\sum_{l=1}^{L}w_{i,l,k}}$$

(32) $$\sigma_{k}^{2,(s+1)}=\frac{\sum_{i=1}^{M}\sum_{l=1}^{L}{\left(z_{i,l}-h_{i,l}\left(x^{\left(s\right)}\right)-\mu_{k}^{\left(s+1\right)}\right)}^{2}w_{i,l,k}}{\sum_{i=1}^{M}\sum_{l=1}^{L}w_{i,l,k}}$$

Then, The detailed workflow for estimating GMM parameters based on MM algorithm is shown in Algorithm 1.
**Algorithm 1** GMM parameters are estimated based on the MM algorithm**Input:** The measurements z;
**Initialize:** Iteration index *s*=0 for MM algorithm;convergence tolerance is δ; and maximum iteration number is $N_{itr}^{\max}$. Given the initial value $\theta^{\left(0\right)}={\left[\pi_{k}^{\left(0\right)},\mu_{k}^{\left(0\right)},{\sigma^{2}}^{\left(0\right)}\right]}^{T}$**MM algorithm loop:**(1)Let s=1 substitut the given initial value $\theta^{\left(0\right)}$ into the iteration Formula (16) to calculate $\theta^{\left(s\right)}$.(2)Substituting $\theta^{\left(s\right)}$ into the iteration Formula (16) to calculate $\theta^{\left(s+1\right)}$.;(3)If the convergence condition $\left|J\left(\theta^{\left(s+1\right)},\left(z-h\left(x\right)\right)\right)-J\left(\theta^{\left(s\right)},\left(z-h\left(x\right)\right)\right)\right|<\delta$ or $s+1=N_{itr}^{\max}$ is satisfied, the MM algorithm is terminated.
**Output:**
$\left\{\pi_{k}^{\left(s+1\right)},{\mu_{k}}^{\left(s+1\right)},\sigma_{k}^{2,(s+1)}\right\}$.


## 5. Algorithm Analysis

### 5.1. Convergence Analysis

Since the MM algorithm is essentially iterative, the following inference is derived from the question of whether this algorithm can guarantee convergence [29].

The convergence of the algorithm refers to the monotonic convergence of the MM algorithm to some stationary point $J^{*}$ of the log-likelihood function J(θ,(z−h(x))). To prove this inference, we first need to prove the detailed workflow of the MM algorithm shown below.

(33) $$J\left(\theta^{\left(s+1\right)};\theta^{\left(s\right)}\right)\geq J\left(\theta^{\left(s\right)};\theta^{\left(s\right)}\right)$$

holds for any $\theta^{\left(s\right)}$ in its parameter space. The proof is as follows: For a given a priori position estimate $x^{\left(s\right)}$, it is easy to show that updates $\pi_{1}^{\left(s+1\right)}$, $\pi_{2}^{\left(s+1\right)}$, ${\mu_{1}}^{\left(s+1\right)}$, ${\mu_{2}}^{\left(s+1\right)}$, $\sigma_{1}^{2,(s+1)}$ and $\sigma_{2}^{2,(s+1)}$ for the Gaussian distribution are global optimal solutions to the corresponding maxi- mization problems. Therefore, we can easily conclude that

(34) $$J\left(\theta_{m}^{\left(s+1\right)},x^{\left(s\right)};\theta^{\left(s\right)}\right)\geq J\left(\theta_{m}^{\left(s\right)},x^{\left(s\right)};\theta^{\left(s\right)}\right)$$

where the right-hand side is exactly $J(\theta^{\left(s\right)};\theta^{\left(s\right)})$.

The new estimate $x^{\left(s+1\right)}$ is obtained by minimizing

(35) $$f\left(x\right)=\sum_{i=1}^{N}\sum_{k=1}^{2}\frac{{\left(z_{i}-h_{i}\left(x\right)-\mu_{k}^{\left(s+1\right)}\right)}^{2}}{\sigma_{k}^{2,(s+1)}}w_{ik}$$

using the BFGS quasi-Newton method with an initial guess set of $x^{\left(s\right)}$. The BFGS quasi-Newton method [30,31,32] guarantees downhill progression toward the local minimum in the Newton step of each iteration, so that the new estimate $\theta^{\left(s+1\right)}$ does not make $J(\theta^{\left(s\right)};\theta^{\left(s\right)})$ decrease in the (s+1)th iteration, that is

(36) $$J\left(\theta_{m}^{\left(s+1\right)},x^{\left(s+1\right)};\theta^{\left(s\right)}\right)\geq J\left(\theta_{m}^{\left(s+1\right)},x^{\left(s\right)};\theta^{\left(s\right)}\right)$$

where the left-hand side is identical to $J(\theta^{\left(s+1\right)};\theta^{\left(s\right)})$. Thus, (36) can be proved. This means that the value of J(θ,(z−h(x))) increases monotonically with iteration. Since it is bounded from above, convergence to some stationary point $J^{*}$ of the J(θ,(z−h(x))) is guaranteed.

### 5.2. Complexity Analysis

Complexity evaluation is often assessed using floating-point operations (FLOPs) as a metric. Floating point arithmetic is a common metric for measuring the amount of computation required to execute an algorithm. It can be used to compare the computational complexity and efficiency of different algorithms. The computational complexity of the MM algorithm under the Gaussian mixture model is shown as follows. We will first define the floating point operations required for some basic operations.

(1) $\varepsilon_{add}$: FLOPs for addition.
(2) $\varepsilon_{sub}$: FLOPs for subtraction.
(3) $\varepsilon_{mul}$: FLOPs for multiplication.
(4) $\varepsilon_{div}$: FLOPs for division.
(5) $\varepsilon_{exp}$: FLOPs for exponents.
(6) $\varepsilon_{pow}$: FLOPs for raising to a real power.
(7) $\varepsilon_{sqrt}$: FLOPs for square roots.

Since the MM algorithm is an iterative algorithm, we analyze the s+1th iteration. Given a priori parameter estimation, the first step of the estimation is to evaluate the $w_{i,l,k}$ of i=1,2,…,M,l=1,2,…,L and k=1,2. This requires a calculation:

(37) $$z_{i,l}-h_{i,l}\left(x^{\left(s\right)}\right)$$

for i=1,2,…,M,l=1,2,…,L.

(38) $$\phi_{i,l,k}=\frac{\pi_{k}^{\left(s\right)}}{\sqrt{2\pi \sigma_{k}^{2,(s)}}}\cdot \exp\left[\frac{-{\left(z_{i,l}-h_{i,l}\left(x^{\left(s\right)}\right)-\mu_{k}^{\left(s\right)}\right)}^{2}}{2\sigma_{k}^{2,(s)}}\right]$$

for i=1,2,…,M,l=1,2,…,L,k=1,2.

(39) $$w_{i,l,k}=\frac{\pi_{k}^{\left(s\right)}N\left(\left(z_{i,l}-h_{i,l}\left(x\right)\right)|\mu_{k}^{\left(s\right)},\sigma_{k}^{2,(s)}\right)}{p(\left(z_{i,l}-h_{i,l}\left(x\right)\right);\theta_{}^{\left(s\right)})}$$

for i=1,2,…,M,l=1,2,…,L,k=1,2. Thus, Equation (37) requires $ML\varepsilon_{sub}$ FLOPs, Equation (38) requires $2(\left(ML+3\right)\varepsilon_{mul}+\left(ML+1\right)\varepsilon_{pow}+ML\varepsilon_{sub}+ML\varepsilon_{div}+ML\varepsilon_{exp})$ FLOPs, and Equation (39) requires $ML(1\varepsilon_{sub}+1\varepsilon_{add}+1\varepsilon_{div})$ FLOPs. Then,

(40) $$\pi_{k}^{\left(s+1\right)}=\frac{\sum_{i=1}^{M}\sum_{l=1}^{L}w_{i,l,k}}{ML}$$

(41) $$\mu_{k}^{\left(s+1\right)}=\frac{\sum_{i=1}^{M}\sum_{l=1}^{L}\left(z_{i,l}-h_{i,l}\left(x^{\left(s\right)}\right)\right)w_{i,l,k}}{\sum_{i=1}^{M}\sum_{l=1}^{L}w_{i,l,k}}$$

(42) $$\sigma_{k}^{2,(s+1)}=\frac{\sum_{i=1}^{M}\sum_{l=1}^{L}{\left(z_{i,l}-h_{i,l}\left(x^{\left(s\right)}\right)-\mu_{k}^{\left(s+1\right)}\right)}^{2}w_{i,l,k}}{\sum_{i=1}^{M}\sum_{l=1}^{L}w_{i,l,k}}$$

for i=1,2,…,M,l=1,2,…,L,k=1,2. It is easy to prove that Equation (40) requires $\left(NK-1\right)\varepsilon_{add}+1\varepsilon_{div}+1\varepsilon_{sub}$ FLOPs, Equation (41) requires $2(NK\varepsilon_{mul}+\left(NK-1\right)\varepsilon_{add}+\varepsilon_{div})$ FLOPs, and Equation (42) requires $2(\left(NK+1\right)\varepsilon_{pow}+NK\varepsilon_{mul}+\left(NK-1\right)\varepsilon_{add}+\varepsilon_{div}+\varepsilon_{sub})$ FLOPs. Define FL(θ) as the total number of FLOPS consumed in one MM iteration to estimate θ. FL(θ) is equal to the total number of FLOPs consumed in Equation (37) through Equation (42), that is

(43) $$\begin{array}{c} FL\left(\theta \right)=\left(6ML-5\right)\varepsilon_{add}+\left(4ML+3\right)\varepsilon_{sub}+\left(6ML+6\right)\varepsilon_{mul}\\ +\left(4ML+4\right)\varepsilon_{pow}+\left(3ML+5\right)\varepsilon_{div}+2ML\varepsilon_{\exp} \end{array}$$

## 6. Simulation

**Figure 1:**
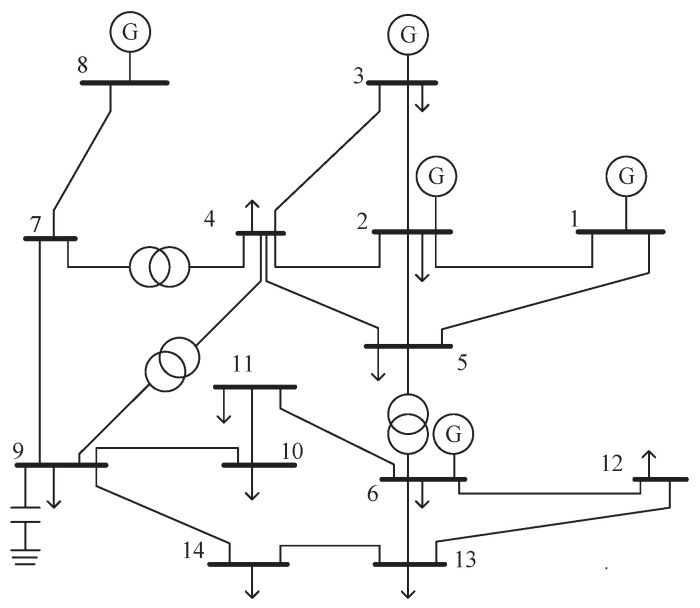
IEEE 14-bus system.

To validate the feasibility of using the MM algorithm to detect outliers, this paper conducts a simulation analysis on the IEEE 14-bus system shown in Figure 1. Based on the relevant data in the Matpower power system simulation package, a conventional power flow calculation is performed, and the obtained system operation data are used as the measurement data of the power system. The simulation parameters utilized throughout the entire simulation process are summarized in Table 1, and the obtained experimental results are shown as follows.

**Figure 2:**
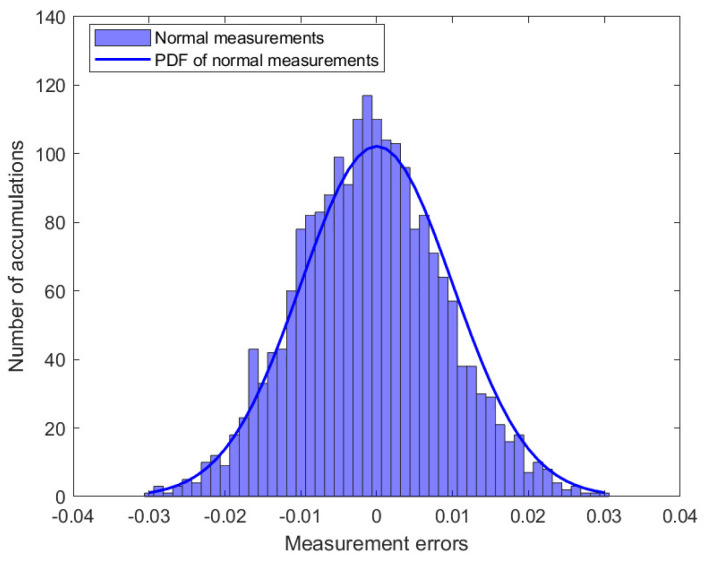
The distribution of measurements errors.

**Figure 3:**
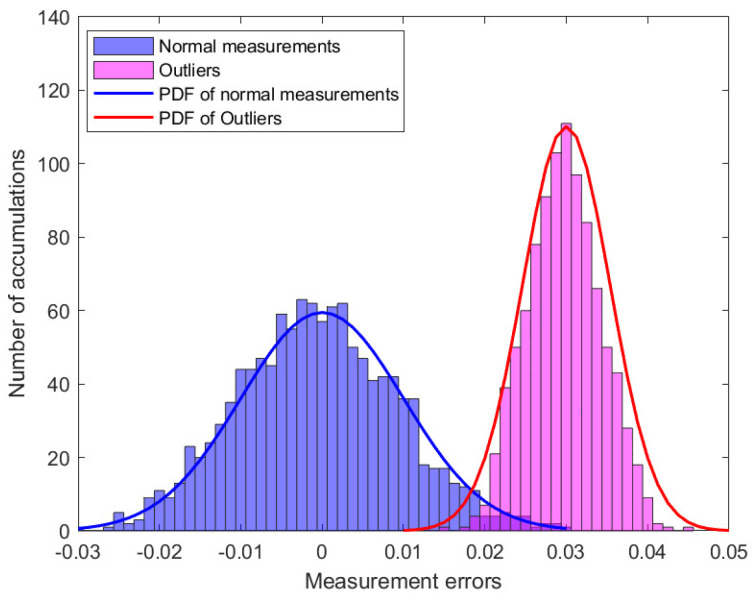
The distribution of measurement errors in the presence of outliers.

**Figure 4:**
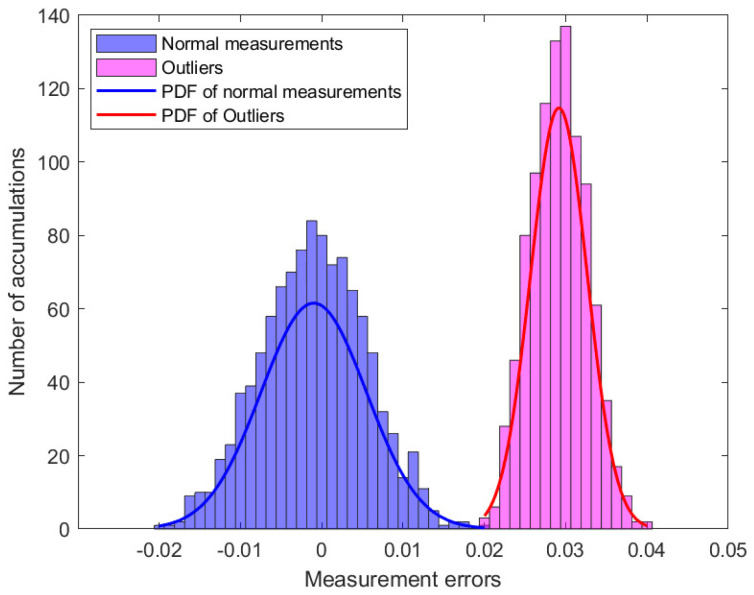
The distribution of measurements errors with MM algorithm.

In this study, simulation experiments containing 50 measurement vectors were analyzed. Figure 2 shows the distribution of the 2050 measurement errors. When there are outliers in the power system, part of the measurement error will change, assuming $\left|a_{i,l}\right|>0$, where $a_{i,l}$ denotes the component of the outlier vector a. Figure 3 shows the distribution of the measurement errors in the presence of outliers. Subsequently, the MM algorithm was used to classify the measurement errors and is presented in Figure 4.

**Figure 5:**
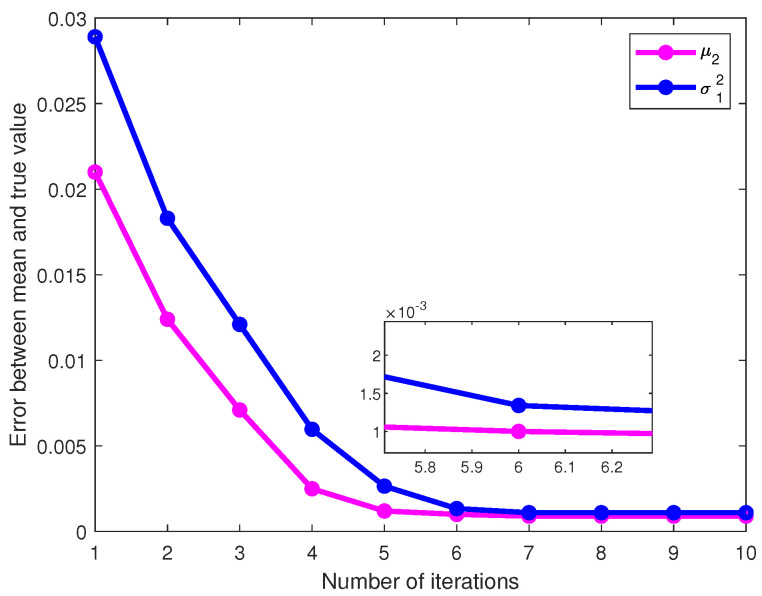
The error of the parameter varies with the number of iterations.

To verify the convergence of the algorithm, it is assumed that M = 41, S = 8, and L = 50. The Monte Carlo method was used to perform 1000 independent experiments to detect the values of parameters $\theta ={\left[\pi_{1},\pi_{2},\mu_{1},\mu_{2},\sigma_{1}^{2},\sigma_{2}^{2}\right]}^{T}$. The errors between the mean of the estimated values and the actual values were calculated for each parameter. As shown in Figure 5, as the number of iterations increases, the estimated mean values of the parameters $\mu_{2}$ and $\sigma_{1}^{2}$ gradually approach the true values, the error decreases, and the values finally converged to 0.00102 and 0.00134 at the sixth iteration, respectively. And, a variation in these errors was observed as the number of buses with outliers increased. The error between the mean and true values of the parameter estimates obtained from 1000 independent experiments was calculated. The equations are given by

(44) $$E\left({\hat{\mu }}_{k}\right)=\left|\frac{\sum_{n=1}^{N}{\hat{\mu }}_{k,n}}{N}-\mu_{k}\right|$$

(45) $$E\left({\hat{\sigma }}_{k}^{2}\right)=\left|\frac{\sum_{n=1}^{N}{\hat{\sigma }}_{k,n}^{2}}{N}-\sigma_{k}^{2}\right|$$

and

(46) $$E\left({\hat{\pi }}_{k}\right)=\left|\frac{\sum_{n=1}^{N}{\hat{\pi }}_{k,n}}{N}-\pi_{k}\right|$$

(47) $$E\left({\hat{\pi }}_{1}\right)=\left|\frac{\sum_{n=1}^{N}{\hat{\pi }}_{1,n}}{N}-\pi_{1}\right|$$

**Figure 6:**
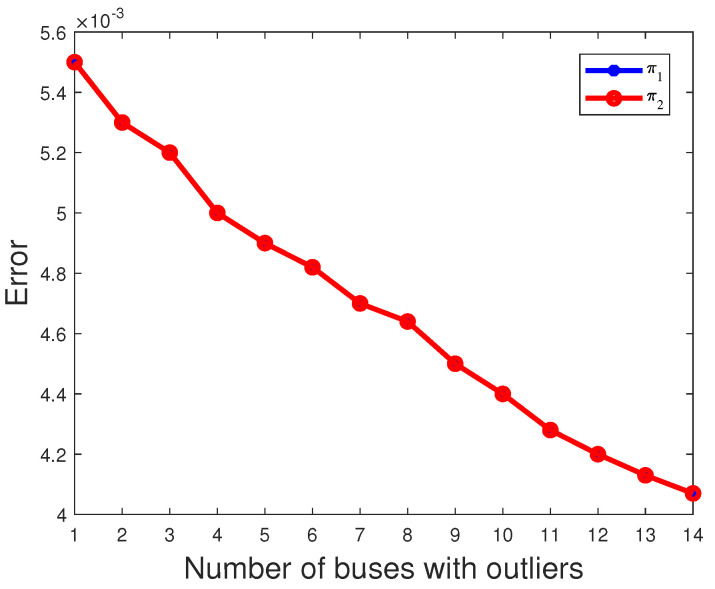
The error of parameter $\pi_{k}$ varies with the number of buses with outliers.

**Figure 7:**
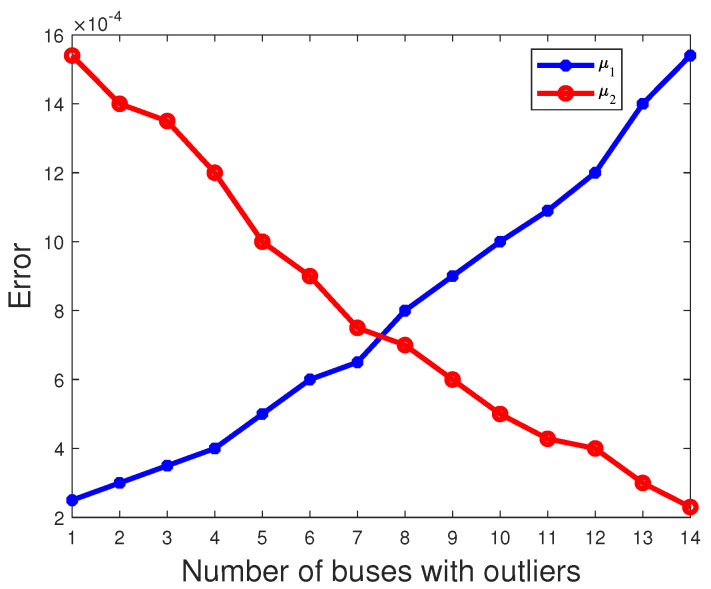
The error of parameter $\mu_{k}$ varies with the number of buses with outliers.

**Figure 8:**
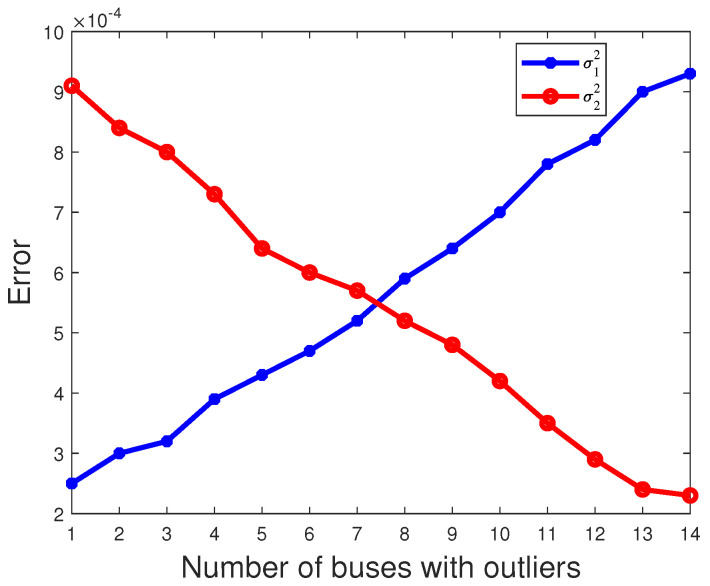
The error of parameter $\sigma_{k}$ varies with the number of buses with outliers.

Since $\pi_{1}>0$,$\pi_{2}>0$ and $\pi_{1}+\pi_{2}=1$, then the error for $\pi_{2}$ can be expressed as

(48) $$E\left({\hat{\pi }}_{2}\right)=\left|\pi_{1}-\frac{\sum_{n=1}^{N}{\hat{\pi }}_{1,n}}{N}\right|$$

where *N* = 1000 and ${\hat{\mu }}_{k,n}$ denotes the estimate of ${\hat{\mu }}_{k}$ obtained in the nth experiment. The estimated mean is then obtained by summing ${\hat{\mu }}_{k,n}$ over 1000 independent experiments and dividing by N. And, due to $E\left(\pi_{1}\right)=E\left(\pi_{2}\right)$, the red line coincides with the blue line in Figure 6. As shown in Figure 6, Figure 7 and Figure 8, it is evident that as the number of buses with outliers gradually increases, we clearly observe a gradual decrease in the error between the estimated mean and the actual values of the parameters $\pi_{2}$, $\mu_{2}$ and $\sigma_{2}$. This is due to the increasing number of outliers as a proportion of the overall measurements. In this scenario, the estimated mean values of the parameters $\pi_{2}$, $\mu_{2}$, and $\sigma_{2}$ gradually increase, getting closer to the true values, which leads to a gradual decrease in the error. In other words, as the number of outlier buses increases, we observe a significant improvement in the accuracy of the parameter estimates.

**Figure 9:**
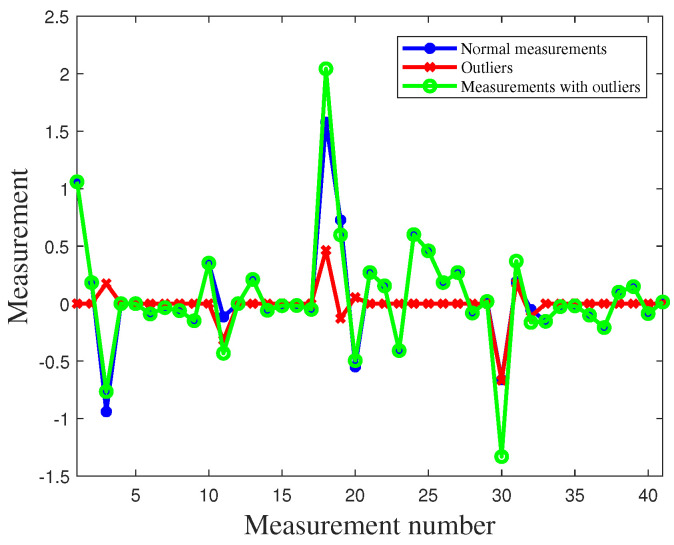
Data distribution before and after outliers in IEEE 14-bus system.

**Figure 10:**
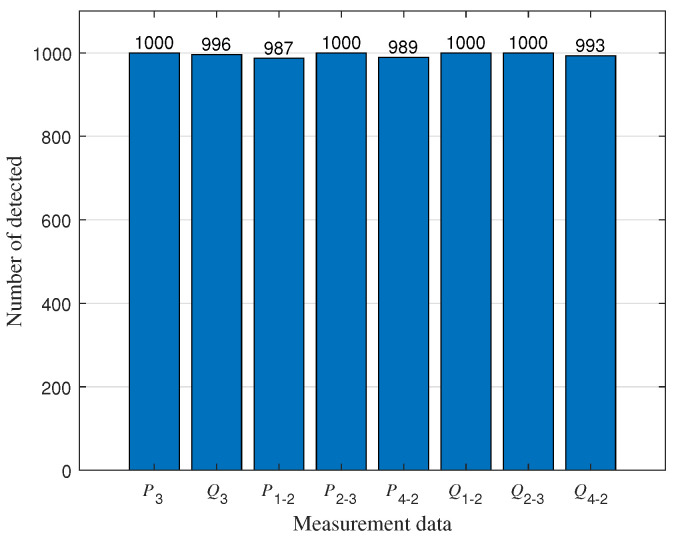
Outlier detection results.

As shown in Figure 9, we assume that outliers exist in the power measurements of $P_{3}$, $Q_{3}$, $P_{1-2}$, $P_{2-3}$, $P_{4-2}$, $Q_{1-2}$, $Q_{2-3}$, and $Q_{4-2}$ among the 41 measurements. To detect outliers, we conducted 1000 independent experiments and plotted Figure 10, showing the results of outlier detection based on the experimental results. By observing Figure 10, we can notice that the measurements in $Q_{3}$,$P_{1-2}$,$P_{4-2}$, and $Q_{4-2}$ are closer to the normal measurements. As a result, a small amount of data has been mistakenly identified as normal data. After applying the MM algorithm for outlier detection, we achieved an extremely high detection rate of over 95%. This implies that the vast majority of outlier values can be accurately detected.

**Figure 11:**
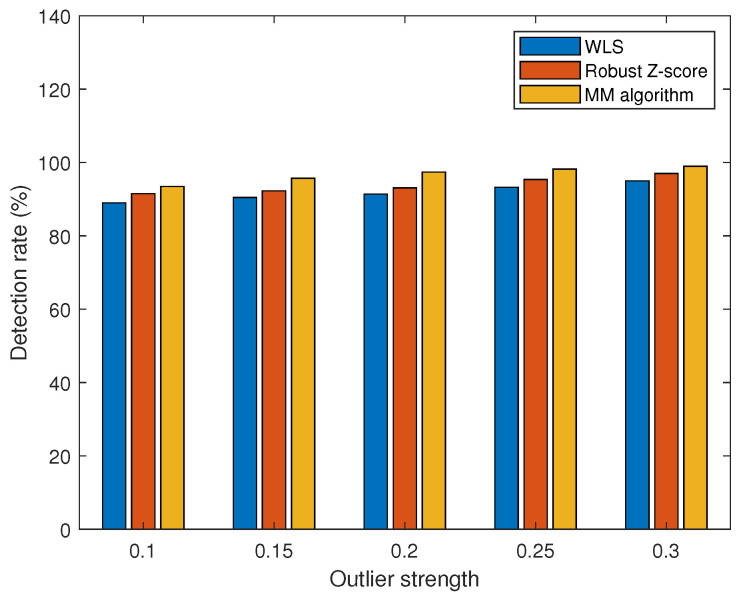
Comparison of detection performance at different outlier strengths.

**Figure 12:**
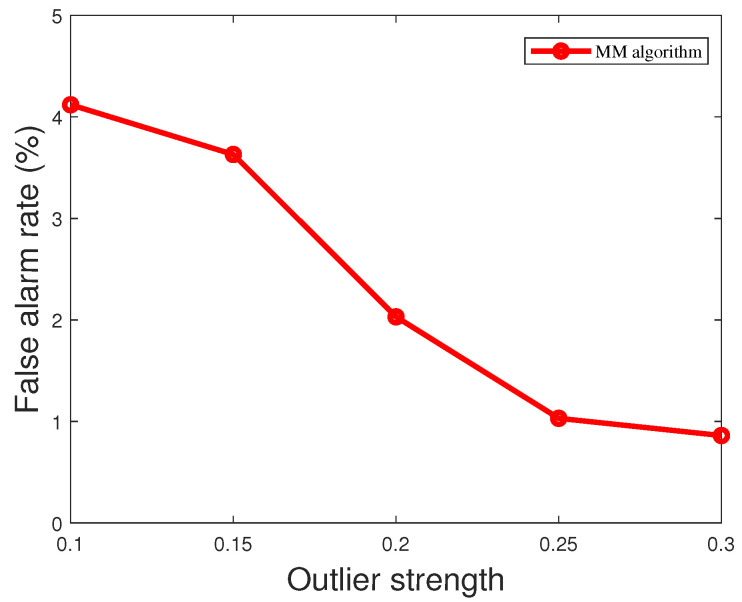
False alarm rate of MM algorithm for different outlier strengths.

To validate the detection performance of the MM algorithm, WLS, and robust Z-score [33] for different outlier strengths, we use the detection rate as a performance metric. Outlier strength is a metric used to quantify the degree of deviation of the outliers from the normal measurements. The outlier strength is defined as

(49) $$\eta =\frac{{∥a∥}_{2}}{{∥z∥}_{2}}$$

where $a\in R^{m\times 1}$ is the outlier vector and $z\in R^{m\times 1}$ is the measurement vector. When the outlier strength is greater, this means that the deviation of the outlier relative to the normal measurement is greater and may have a more significant effect on the system. As shown in Figure 11, when the outlier strength is low, the outliers are not easily detected. However, the MM algorithm proposed in this paper outperforms WLS and robust Z-score in terms of detection performance. This is due to the iterative optimization process of the MM algorithm, which can gradually approach the optimal solution and shows good convergence, stability, and robustness in practice. Since the MM algorithm uses probabilistic models, it is better able to deal with outliers in the data and to reduce the impact of outliers on the results. The MM algorithm can limit the impact of outliers and provide more accurate parameter estimation, resulting in better performance in outlier detection. In contrast, WLS may suffer from outliers, leading to biased parameter estimates. The robust Z-score is more robust to outliers relative to the traditional Z-score, but MM algorithms are usually better at robustness to outliers in the modeling process. As the strength of the outliers varies, the false alarm rate also varies. As shown in Figure 12, the greater the strength of the outliers, the easier the outliers are detected and the smaller the false alarm rate. When the strength of the outliers is greater than 0.3, the false alarm rate decreases to less than 1%.

## 7. Conclusions

Considering the impact of the presence of outliers on security in power systems, the introduction of MM algorithms for outlier detection and localization is an effective approach. The algorithm clusters and models the measurement errors using GMM and solves the model parameters iteratively using the MM algorithm. The feasibility and convergence of the MM algorithm in power systems are investigated through a simulation analysis on an IEEE 14-bus system, and the performance is compared with the WLS and robust Z-score at the same outlier intensity. The experimental results show that the MM algorithm can efficiently identify outliers in the power system and its detection rate can reach 95%. The results of this research emphasize the superiority of the MM algorithm in power system outlier detection. Compared with WLS and robust Z-score, the MM algorithm can model and detect outliers more accurately by introducing a probabilistic model and an iterative optimization algorithm to improve the security and reliability of the power system.

## Figures and Tables

**Table 1:** Simulation parameters.

Parameter	Value
*M*	41
$\pi_{1}$	0.8
$\pi_{2}$	0.2
$\mu_{1}$	0
$\mu_{2}$	0.03
$\sigma_{1}^{2}$	0.01
$\sigma_{2}^{2}$	0.0025
Δ	$10^{-6}$
$N_{itr}^{\max}$	100

## Data Availability

Not applicable.
